# Structural Evolution of Biomedical Microrobots: From Rigid to Soft to Rigid–Soft Integrated Systems

**DOI:** 10.3390/gels12090803

**Published:** 2026-09-02

**Authors:** Gang Wang, Hongfei Liang, Xuefei Liu, Wenjun Xiao, Degui Wang, Jinshun Bi, Abuduwayiti Aierken, Mingqiang Liu, Ziqiang Xu, Changsong Gao, Zhen Wang, Yan Wu

**Affiliations:** 1 School of Physics and Electronic Science, Guizhou Normal University, Guiyang 550025, China; 2 School of Integrated Circuit, Guizhou Normal University, Guiyang 550025, China

**Keywords:** microrobots, structural evolution, rigid structures, soft structures, rigid–soft integrated structures, biomedical applications

## Abstract

Owing to their small size, micron-scale accuracy, rapid response, and biocompatibility, microrobots are emerging as a promising tool for biomedical applications, especially in targeted drug delivery and minimally invasive microsurgery. The architectural configuration of microrobots governs their locomotion performance, environmental adaptability, and functional integration capacity. Although existing reviews have systematically organized this field by actuation strategies, material categories, or application scenarios, a comprehensive summary centered on the structural evolution paradigm remains conspicuously absent. This review aims to fill this gap by systematically tracing the evolutionary trajectory of microrobot structures from rigid architectures through soft configurations to rigid–soft integrated systems. First, the foundational principles underlying structural evolution were introduced, encompassing the connotation of structure, fluid dynamics constraints, and the drivers of structural innovation. Then, rigid microrobot architectures were systematically elucidated, including geometric asymmetric and surface asymmetric designs. Subsequently, soft microrobot structures, covering both predefined deformation structures and dynamically reconfigurable architectures, were discussed. Finally, the rigid–soft integrated systems reconciling compliance and performance through spatial heterogeneity or temporal stiffness modulation were systematically surveyed. This evolutionary trend reflects a transition from optimizing individual performance parameters toward achieving balanced functional synergy across distinct task phases. Based on the current research progress, this review also presents future research directions in data-driven structural optimization, reconfigurable architectures, and autonomous structural intelligence, offering strategic guidance for next-generation microrobot design.

## 1. Introduction

Precision medicine fundamentally aims to achieve safe and efficient in vivo delivery of therapeutic agents to specific biological targets, followed by post-intervention biodegradation or clearance mechanisms. However, conventional drug delivery is inherently constrained by passive stochastic transport within the bloodstream and elevated interstitial fluid pressure characteristic of tumor tissues. Consequently, the actual drug accumulation at target tumor cells is typically much lower than the injection dose, and the remaining drug circulating in vivo often leads to significant off-target toxicity toward normal cells [1,2]. The advent of microrobots offers a paradigm-shifting solution to address these critical biomedical challenges [3,4,5]. Microrobots refer to miniature robotic systems with geometric dimensions spanning several micrometers to hundreds of micrometers that achieve autonomous locomotion in biological fluids through external field actuation or chemical self-propulsion [6,7,8,9]. In contrast to macroscopic robots, microrobots operate in low-Reynolds-number environments where viscous forces dominate over inertial effects. As a result, any reciprocal motion sequence produces no net displacement, a constraint known as the Scallop Theorem [10,11,12,13]. Consequently, propulsion necessarily depends on breaking time-reversal symmetry via structural asymmetry or non-reciprocal deformation. To satisfy this physical imperative, diverse actuation strategies, including magnetic, optical, acoustic, electrical, and chemical fields [14,15], have been coupled with material categories spanning metals, polymers, hydrogels, inorganic oxides, and biohybrid systems [16,17]. Each combination imposes distinct requirements on structural configuration and fabrication protocols.

To date, several reviews have systematically organized the microrobotics field from the perspectives of actuation modalities [14,15], material categories [16,17], manufacturing technologies [18], and application scenarios [19,20,21]. The microrobotics roadmap published in 2026 provides comprehensive coverage from a multidimensional perspective [22]. Interdisciplinary topics, including AI-integrated microrobots, in vivo manipulation frontiers, and environmental and health impacts, have also been systematically reviewed [23,24,25]. However, these works predominantly treat structural configuration as a derivative of material or actuation choices, rather than as an independent design dimension that orchestrates these elements into task-specific functions. In fact, the functional performance of microrobots, whether concerning targeting efficiency, tissue penetration capability, or in vivo safety, is determined primarily by structural mechanical design rather than solely by actuation modality or material selection [26,27,28]. Consequently, an overarching framework that traces how microrobot architectures have evolved remains absent. This review aims to address this gap by establishing structural evolution as the primary analytical axis. Furthermore, in biomedical applications, microrobots can be used for targeted drug delivery, minimally invasive surgery, cellular manipulation, and bioimaging [29,30]. However, these tasks impose stringent and often contradictory mechanical demands across multiple stages, with the navigation phase requiring high compliance for safe passage through tortuous and constricted vascular networks, the penetration phase demanding adequate stiffness to breach tissue barriers, and the execution phase necessitating stable positioning for precise payload release or therapeutic action. To meet these multifaceted requirements, structural design acts as the pivotal link that integrates actuation and material capabilities into task-specific functions. In other words, materials prescribe the available functionality, actuation imparts motility, and structural design orchestrates these constituents into reliable, task-specific biomedical functions.

**Figure 1:**
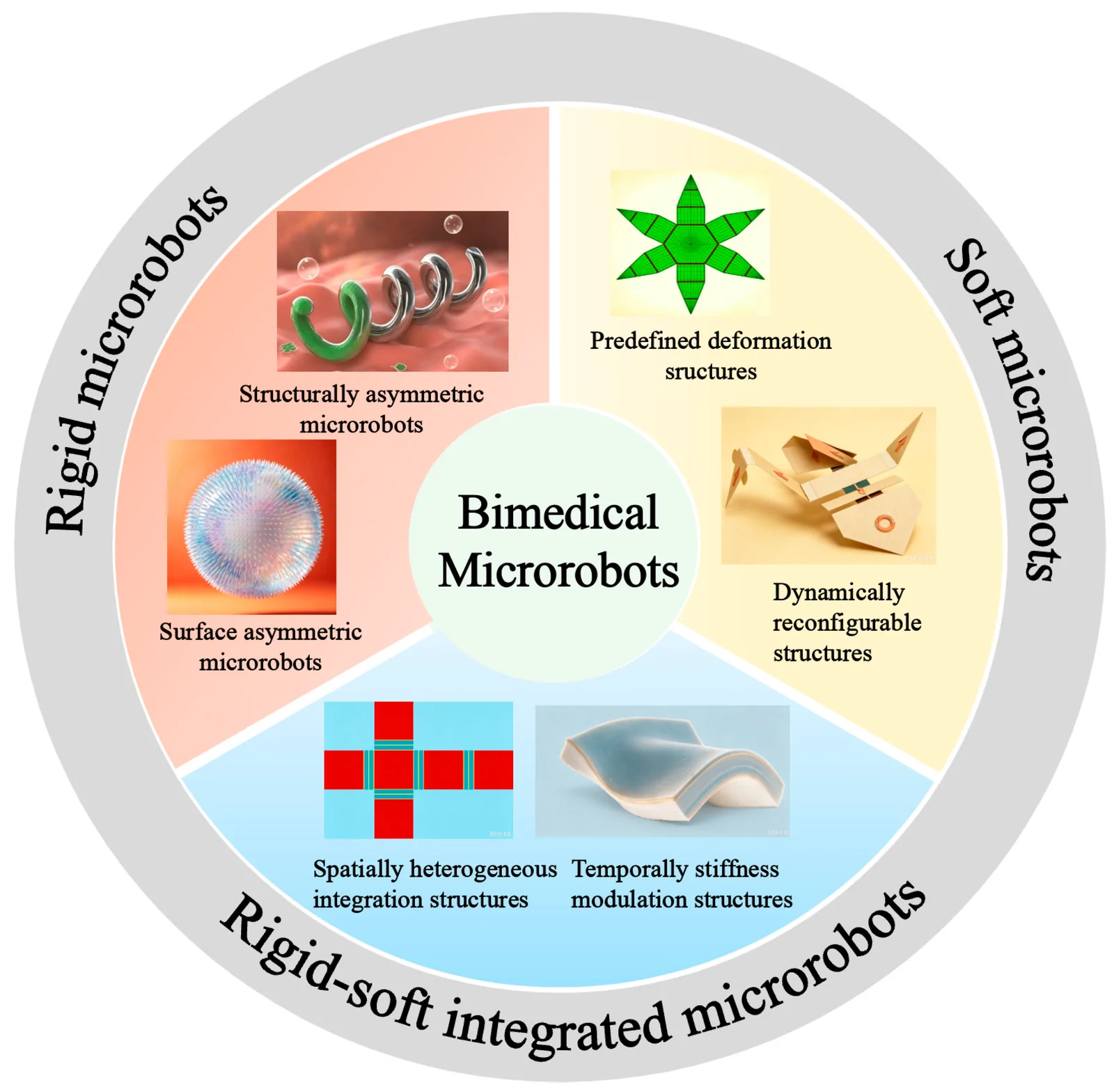
A schematic showing the outline of this review, including rigid microrobots, soft microrobots, and rigid–soft integrated microrobots.

Grounded in this premise, the present review adopts the evolutionary trajectory of microrobot structures as its organizing framework and systematically surveys the progressive maturation of three structural paradigms, specifically rigid, soft, and rigid–soft integrated architectures. Despite substantial temporal concurrence among these categories, their sequential emergence nevertheless embodies an intrinsic logical evolution. Early microrobots predominantly employed rigid architectures constructed from high-modulus materials such as metals or photoresists, exploiting fixed geometric or surface asymmetry to achieve deterministic locomotion in low-Reynolds-number fluids [31]. While rigid structures offer precise control and high propulsion efficiency, their intrinsic stiffness, typically in the GPa range, creates severe mechanical mismatches with soft biological tissues, risking tissue injury, compromising environmental adaptability, and limiting navigability through narrow lumens [32]. In response, soft structures utilizing hydrogels, elastomers, and stimuli-responsive polymers have emerged, harnessing programmable deformation to enhance biomechanical compatibility, reduce interfacial stress, and navigate complex anatomical geometries [33]. Nevertheless, soft structures tend to exhibit reduced force output and motion precision compared with rigid counterparts of comparable dimensions, posing a critical bottleneck for tasks requiring mechanical interaction with dense tissues. This intrinsic rigidity-compliance trade-off has catalyzed the development of rigid–soft integrated architectures that seek a dialectical synthesis through spatiotemporal programming of mechanical properties, synergistically combining the actuation precision of rigid components with the environmental adaptability of soft constituents [34,35]. It should be noted that this trade-off is not universal, because architecture, material reinforcement, actuation mechanism, geometric scale, and operating environment collectively determine the achievable force, strain, and positioning accuracy. Accordingly, this evolutionary trajectory reflects the progressive refinement of microrobot structural design in response to increasingly authentic biomedical demands. The literature search and review methodology is detailed in Appendix A, and the overall structure of this review is depicted in Figure 1.

## 2. Foundational Principles Underlying Structural Evolution of Microrobots

### 2.1. Connotation of Structure

The structure as discussed in this review encompasses two hierarchical levels. The first level refers to macroscopic geometric configuration and surface physicochemical properties, namely the overall three-dimensional (3D) shape of the microrobot and the spatial distribution of its surface properties. The second level concerns the microscopic spatial distribution of materials, including homogeneous structures, multi-material heterogeneous structures, and structures with continuous gradients in crosslinking density or composition. The elastic modulus serves as the most direct quantitative metric for distinguishing the three structural paradigms. Rigid microrobots typically exhibit elastic moduli on the order of GPa, whereas soft microrobots are characterized by moduli in the kPa to MPa range [32], corresponding to a material-level difference spanning approximately 3–6 orders of magnitude. However, elastic modulus alone is insufficient for rigorous classification, as the effective structural stiffness of a microrobot also depends on its geometry, dimensions, loading mode, and boundary conditions. Accordingly, the classification adopted herein integrates four criteria, namely constituent material modulus, effective structural stiffness under operational loading, maximum reversible deformability, and the functional role of each component. Rigid microrobots are defined as systems in which high material modulus and structural configuration jointly yield deterministic locomotion with minimal elastic deformation. Soft microrobots are characterized by low material modulus and high deformability, where shape adaptation is functionally integral to propulsion or environmental interaction. Rigid–soft integrated systems deliberately combine high- and low-modulus components in discrete spatial domains, assigning rigid segments to force transmission and actuation precision while employing soft segments for compliance and interfacial stress reduction. Borderline cases, such as phase-change materials whose modulus spans GPa and MPa regimes depending on thermal state, and high-modulus materials configured as origami-inspired designs, are classified according to their dominant functional behavior and the design intent governing their structural compliance rather than solely by material modulus. Notably, the elastic modulus of biological tissues spans a broad range from endothelial cells (approximately 1–10 kPa) to bone tissue (approximately 15–20 GPa) [6]. This wide span implies that microrobots with a single homogeneous structure can hardly achieve both safe contact and effective manipulation simultaneously across all physiological environments, necessitating either multi-material designs or adaptive mechanical structures.

### 2.2. Constraints of Low-Reynolds-Number Fluid Dynamics

When a microscale robot moves in biological fluids, its characteristic Reynolds number (Re = ρvL/μ) typically satisfies Re ≪ 1. Under this condition, fluid inertia is entirely negligible, and the time-reversal symmetry of the Stokes equation dictates that any reciprocal motion sequence with time-reversal symmetry produces zero net displacement, which is known as the Scallop Theorem [11-131212]. This theorem imposes the most fundamental physical constraint on all structural paradigms that the propulsion of microrobots necessarily depends on breaking time-reversal symmetry in its kinematics. Rigid microrobots achieve this through fixed asymmetric geometric configurations or surface asymmetry, soft microrobots do so by generating asymmetric deformation waves, and rigid–soft integrated microrobots allow different symmetry-breaking mechanisms to be invoked at different stages of a task. It is worth noting that the helical rotating propulsion of bacterial flagella in nature serves as a classic example of breaking the scallop theorem. The hydrodynamic analysis of this mechanism has provided an important theoretical foundation for the structural design of artificial microrobots [36].

### 2.3. Intertwined Factors Driving Structural Evolution

The reasons propelling the structural evolution of microrobots arise from three intertwined aspects. First, the multi-stage mechanical demands of biomedical applications play a crucial role. A typical targeted drug delivery mission can be decomposed into three phases, namely navigation, penetration, and execution. The navigation phase requires high compliance for safe passage through tortuous lumens, the penetration phase demands high stiffness to breach tissue barriers, and the execution phase necessitates stable positioning for precise payload release. These three requirements constitute a fundamental contradiction within a single homogeneous structure. Second, continuous breakthroughs in materials science also contribute to this evolution. For instance, magnetic materials offer advantages including remote controllability, non-contact operation, and strong tissue penetration capability, although their long-term safety depends on composition, dosage, field, and clearance mechanisms [37,38,39]. The emergence of smart soft materials, such as stimuli-responsive hydrogels and liquid crystal elastomers, has endowed microrobots with active deformation capabilities [40,41,42]. Phase-change materials and magnetorheological materials enable microrobots to achieve controllable stiffness switching [43]. Third, iterative upgrades in manufacturing technologies also drive this evolution. For example, template-assisted electrodeposition techniques enabled the precise fabrication of metallic nanorods and helical structures [44], two-photon polymerization technology realized the direct printing of arbitrary 3D polymer structures at a resolution of approximately 100 nm [45,46,47,48], and the rise of 4D printing further inscribed deformation pathways in the time dimension into the material during the manufacturing process [49,50,51,52,53].

### 2.4. Conceptual Nature of Structural Evolution

It is essential to clarify that the term “evolution” as used throughout this review denotes a conceptual and functional paradigm shift in design philosophy rather than a strictly chronological or mutually exclusive historical progression. The three structural paradigms, namely rigid, soft, and rigid–soft integrated, are not successive technological replacements but coexisting design philosophies that have matured unevenly over time. For instance, soft hydrogel-based actuators were demonstrated as early as the 2000s, whereas many rigid helical microswimmers were developed later in the 2010s. Accordingly, the term “structural evolution” in this review does not imply a strict chronological sequence. Instead, it refers to a conceptual and functional evolution in design priorities, moving from an initial emphasis on deterministic locomotion and precision in rigid systems, through a pursuit of environmental adaptability and biocompatibility in soft systems, to the current integration of performance and compliance within unified rigid–soft integrated systems. This conceptual framing accounts for the temporal overlap among paradigms and clarifies that evolution concerns changing design philosophies and functional integration rather than a strictly sequential historical timeline. 

## 3. Rigid Microrobots

Rigid microrobots are constructed from high-elastic-modulus materials, such as metal and SU-8 photoresist. These structures operate by trading geometric asymmetry for non-reciprocal motion in low-Reynolds-number environments to achieve net locomotion. The motion capability of rigid microrobots derives from either the asymmetry of geometric structures (e.g., helical shapes) or the anisotropy of surface properties (e.g., Janus structures with heterogeneous surface composition). Based on how asymmetry is realized, the structural design of rigid microrobots can be categorized into geometric asymmetric and surface asymmetric types. Geometric asymmetric microrobots feature macroscopic geometric asymmetry (e.g., helical shapes) that enable net locomotion in fluids, whereas surface asymmetric types maintain symmetric macroscopic structures (e.g., spherical shapes) and their locomotion relies on asymmetric distribution of substances on the microrobot surface or an asymmetric fluid environment.

### 3.1. Geometric Asymmetric Structures

Geometric asymmetric microrobots achieve propulsion through the inherent asymmetry of their overall 3D geometry. The geometric design of such structures directly determines both motion patterns and propulsion efficiency, requiring fabrication processes capable of precisely replicating predefined 3D shapes. Based on the characteristics of the geometric configuration, geometric asymmetric microrobots can be classified into helical structures, rod/wire-like structures, tubular structures, and other asymmetric configurations.

**Figure 2:**
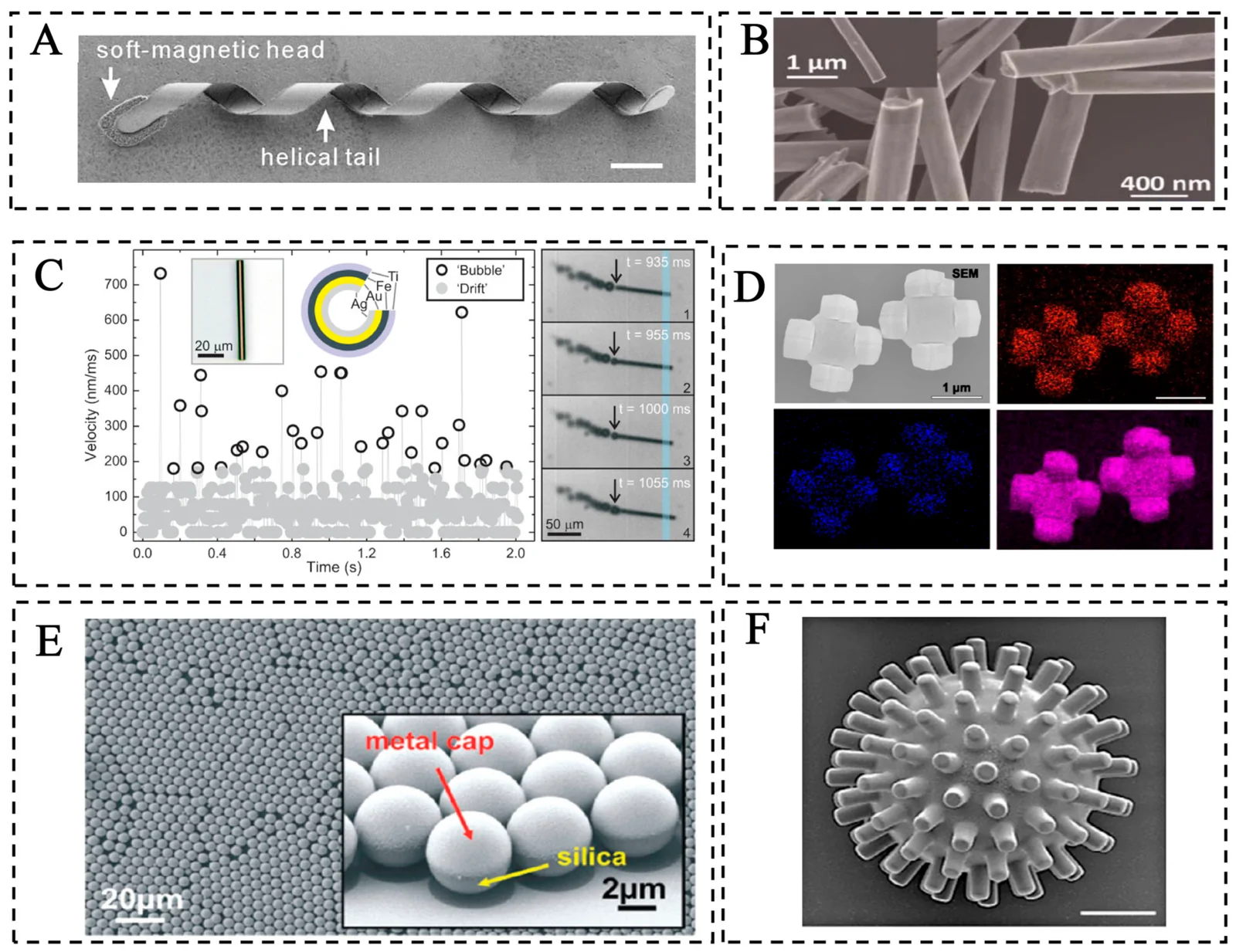
The rigid microrobots. (**A**) The helical microrobots (reprinted from Ref. [54] Copyright © 2009, American Institute of Physics). (**B**) The rod-like microrobots (reprinted from Ref. [86] Copyright © 2012, American Chemical Society). (**C**) The tubular microrobots (reprinted from Ref. [94] Copyright © 2008, Wiley-VCH GmbH). (**D**) The gear-like microrobots (reprinted from Ref. [96] Copyright © 2026, Springer Nature Limited). (**E**) The Janus microrobots (reprinted from Ref. [102] Copyright © 2012, American Chemical Society). (**F**) The spherical surface walker (reprinted from Ref. [67] Copyright © 2021, Wiley-VCH GmbH).

The helical structure is the most representative configuration among the asymmetric structures and is widely recognized as the most important design in rigid microrobot research. Inspired by the helical geometry of bacterial flagella in nature, the core geometric feature of helical microrobots is a continuous helical surface with chirality. The direction of left or right rotation directly determines the propulsion orientation. Under the action of a rotating magnetic field, the helix rotates around its long axis, and through viscous coupling with the surrounding fluid, the rotational motion is converted into translational motion along the axis. The research on helical microrobots has evolved from achieving basic mobility to developing specific functionalities and pursuing biological applicability. In the early stage of research, researchers mainly focused on the fabrication of microrobots, as well as the stability and controllability of their locomotion in low-Reynolds-number fluid environments [54,55,56,57,58,59,60,61]. For instance, Zhang et al. used the self-scrolling technique to fabricate helical microrobots named artificial bacterial flagella (ABF) for the first time, and successfully achieved their controllable locomotion under a rotating magnetic field (Figure 2A) [54]. Technologies such as glancing angle deposition (GLAD), template-assisted electrodeposition, and 3D direct laser writing (DLW) have been employed for microrobot fabrication [62,63,64,65]. It is worth noting that DLW has stood out as a compelling fabrication technique in recent years, by virtue of its high resolution, miniaturized feature sizes, and unrestricted geometric versatility [66,67,68]. Subsequently, researchers center on functional capabilities such as object transportation or grasping in vitro. Various microrobot configurations, including grippers, syringe-like architectures and screw-pump structures, have been proposed [69,70,71,72,73]. For example, Sánchez et al. employed sperm-carrying helical micromotors to enhance sperm motility for the treatment of asthenozoospermia [69]. Huang et al. employed a screw pump microrobot for helical microswimmer transport tasks [70]. Later, researchers have devoted considerable effort to addressing the challenges of biocompatibility, imaging capability, and degradability encountered by microrobots in biological environments, and have proposed a variety of solutions [74,75,76,77,78,79,80,81]. For instance, by coating the surface with Ti, the rigid microrobots have demonstrated improved cytocompatibility in in vitro assays, though comprehensive in vivo biocompatibility assessment remains necessary [74,75]. Imaging methods such as fluorescence imaging [76,77,78], magnetic resonance imaging (MRI) [79,80], and acoustic imaging [81] have been proposed to address the in vivo imaging issues within the microrobots.

Rod/wire-like structures are characterized by quasi-one-dimensional geometries with high aspect ratios [82,83,84,85,86,87,88]. Their propulsion capability originates from geometric anisotropy rather than chirality that when placed in an oscillating acoustic field or a rotating magnetic field, the difference in fluid drag along the long and short axes of the slender body leads to an asymmetric flow field distribution, generating a net propulsive force along the long axis. For instance, Wang et al. demonstrated that such nanorods utilize their geometric asymmetry to achieve acoustophoretic propulsion in an ultrasound field (Figure 2B) [86]. Zhang et al. showed that rotating Ni nanowires achieve controlled propulsion and cargo transport near patterned surfaces [87]. The magnetically actuated multi-link nanoswimmer developed by Jang et al. generates undulatory propulsion under a rotating magnetic field, expanding the motion degrees of freedom of rod-like structures [88]. In biomedical applications, nanorod structures have been employed in scenarios such as circulating tumor cell capture and biosensing, owing to their high specific surface area and ease of functionalization.

Tubular structures are characterized by quasi-two-dimensional rolled-up geometries featuring an internal cavity and at least one opening [89,90,91,92,93,94,95]. Their propulsion relies on the directional confinement by the cavity of bubbles or fluid jets generated by chemical reactions that catalytic reactions occur on the inner surface of the cavity, and bubbles constrained by the tube wall can only be ejected from the opening direction, generating net propulsive force via momentum recoil. For example, Solovev et al. utilized the thin-film stress mismatch principle to fabricate Ti/Fe/Au microtubes with diameters in the range of 5–10 µm, achieving propulsion speeds of up to approximately 50 body lengths per second in hydrogen peroxide solution [93]. Mei et al. reported self-rolled-up titanium dioxide/platinum microtube robots, enabling precise control of tube diameter and length by adjusting the number of rolled layers (Figure 2C) [94]. The advantage of this configuration is that bubble-induced thrust far exceeds phoretic propulsion, yielding extremely high propulsion efficiency. However, the requirement for hydrogen peroxide as a chemical fuel, along with its associated cytotoxicity concerns at present levels, represents a major obstacle for in vivo biomedical applications [95].

Other asymmetric structures include gear-like, star-like, and other configurations with asymmetric geometric features [96,97,98,99,100]. Gear-like structures exploit rotational symmetry in their design for specialized manipulation tasks. For example, Liu et al. developed magnetically controlled microwheel robots based on metal–organic frameworks (MOFs), whose architecture enables multiple switchable motion modes, including standing, lying, rotation, axial rolling, and radial rolling under a three-dimensional Helmholtz coil system, demonstrating excellent magnetic actuation and precise maneuverability across various biological media (Figure 2D) [96]. In terms of star-like configurations, He et al. designed star-like magnetic sheet robots with a thickness of approximately 40 μm that can freely navigate on water surfaces and achieve capture, transport, and release of non-magnetic microscale objects by modulating the frequency and orientation of a rotating magnetic field [99].

### 3.2. Surface Asymmetric Structures

Surface asymmetric microrobots are highly symmetric in their macroscopic 3D geometry (e.g., spherical), with asymmetry originating from a gradient distribution of surface physicochemical properties or boundary layer fluid effects induced by proximity to a substrate. Based on the source of asymmetry, they can be subdivided into structures with asymmetric distribution of catalytic active substances on the microrobot surface and structures that exploit boundary layer effects near substrate surfaces for propulsion.

Structures with asymmetric distribution of catalytic active substances on the robot surface are typified by Janus spheres [101,102,103,104,105]. Half the surface of such spherical particles is coated with a catalyst (e.g., platinum, gold, manganese dioxide), while the other half is an inert surface. In solutions containing chemical fuel, the chemical reaction on the catalytic side generates concentration gradients or bubbles, propelling the particle in a directed manner through self-diffusiophoresis or bubble recoil forces. For instance, Howse et al. reported the self-diffusiophoretic motion of Janus platinum-polystyrene spheres in hydrogen peroxide solution [101]. Baraban et al. fabricated Pt/SiO_2_ Janus microspheres with self-phoretic propulsion in hydrogen peroxide solution (Figure 2E) [102]. Gao et al. further developed Janus gold–manganese dioxide spherical microrobots, utilizing manganese dioxide to catalyze hydrogen peroxide and generate oxygen bubbles for rapid propulsion [103].

Structures requiring proximity to a substrate surface to exploit fluid asymmetry on the substrate side for motion include rotors, nanowires, spheres, spherical chains, etc. [67,106,107,108,109,110,111,112,113]. These microrobots are also known as surface-rolling microrobots. Under magnetic field control, they roll forward on surfaces by leveraging fluid interactions between themselves and the adjacent substrate. For instance, Wang et al. developed a spherical surface walker with engineered surface microstructures, demonstrating a fourfold increase in velocity (507 μm/s) on patterned substrates compared to its smooth counterpart (Figure 2F) [67]. Sing et al. demonstrated that self-assembled colloidal chains could serve as surface walkers under rotating magnetic fields, where the proximity to the substrate broke the time-reversal symmetry and generated controllable surface-induced flows for targeted cargo transport (Figure 2F) [109]. However, their motion is confined to the substrate surface or its immediate vicinity. Once they enter free fluid, they generally fail to produce effective displacement. In biomedical applications, these structures exhibit a unique motion enhancement effect in near-wall regions such as blood vessel walls or tissue interfaces, which can be leveraged to improve the targeting efficiency of microrobots at biological interfaces.

### 3.3. Advantages and Limitations of Rigid Microrobots

After nearly two decades of research, rigid microrobots have established a relatively comprehensive system encompassing design, fabrication, actuation, and characterization. Their core advantages lie in precise and controllable movement as well as high motion speed. Additionally, their theoretical models, including low-Reynolds-number fluid mechanics and magnetic mechanics, can well describe their movement behaviors.

However, the rigidity of the structure also reveals its drawbacks, as the inflexible structure lacks the ability to adapt to the physiological environment. As research indicates, traditional rigid robots have disadvantages, including limited flexibility of motion and poor adaptability to the environment. When encountering a narrow lumen with a diameter smaller than itself, the rigid microrobot can only face a binary outcome of either passing through or becoming blocked, and it cannot actively deform to adapt to spatial constraints. The mechanical mismatch between rigid materials (with elastic modulus in the GPa range) and soft biological tissues can lead to damage during interaction [114]. Additionally, biocompatibility and biodegradation remain important considerations for in vivo applications. These inherent limitations have driven researchers to explore soft structures that offer better environmental adaptability and safer tissue interaction.

## 4. Soft Microrobots

The fundamental design shift of soft microrobots lies in replacing the fixed asymmetry of rigid structures with the programmable deformation capability of low-modulus (kPa to MPa range) materials, such that motion originates from the active deformation of the structure under external stimuli [115,116,117,118,119,120,121,122,123,124,125,126,127,128,129,130,131,132,133,134,135,136,137,138,139,140,141,142,143,144,145,146]. Based on whether the deformation mode can be altered online during operation, soft structures can be divided into two categories, namely deformation-driven structures with predefined deformation patterns and those with reconfigurable deformation patterns. For predefined deformation structures, the deformation mode is determined by the spatial distribution and material properties established during the fabrication stage, and environmental stimuli merely trigger a predefined deformation pathway. In contrast, for dynamically reconfigurable structures, the deformation mode can be switched online during operation by adjusting the parameters of the external actuating field, such as magnetic field orientation and intensity.

### 4.1. Predefined Deformation Structures

The deformation mode of predefined deformation structures is primarily determined by the material properties and geometric configuration established during the fabrication stage. The design core lies in encoding deformation pathways into the material combinations and internal structure. Based on structural geometric features, existing designs include bilayer/multilayer beam deformation structures, single-layer beam structures, and origami/kirigami structures. The deformation pathways of these structures are largely fixed by their initial design, meaning that environmental stimuli can only trigger predetermined shape changes and cannot enable online reconfiguration of the deformation mode.

**Figure 3:**
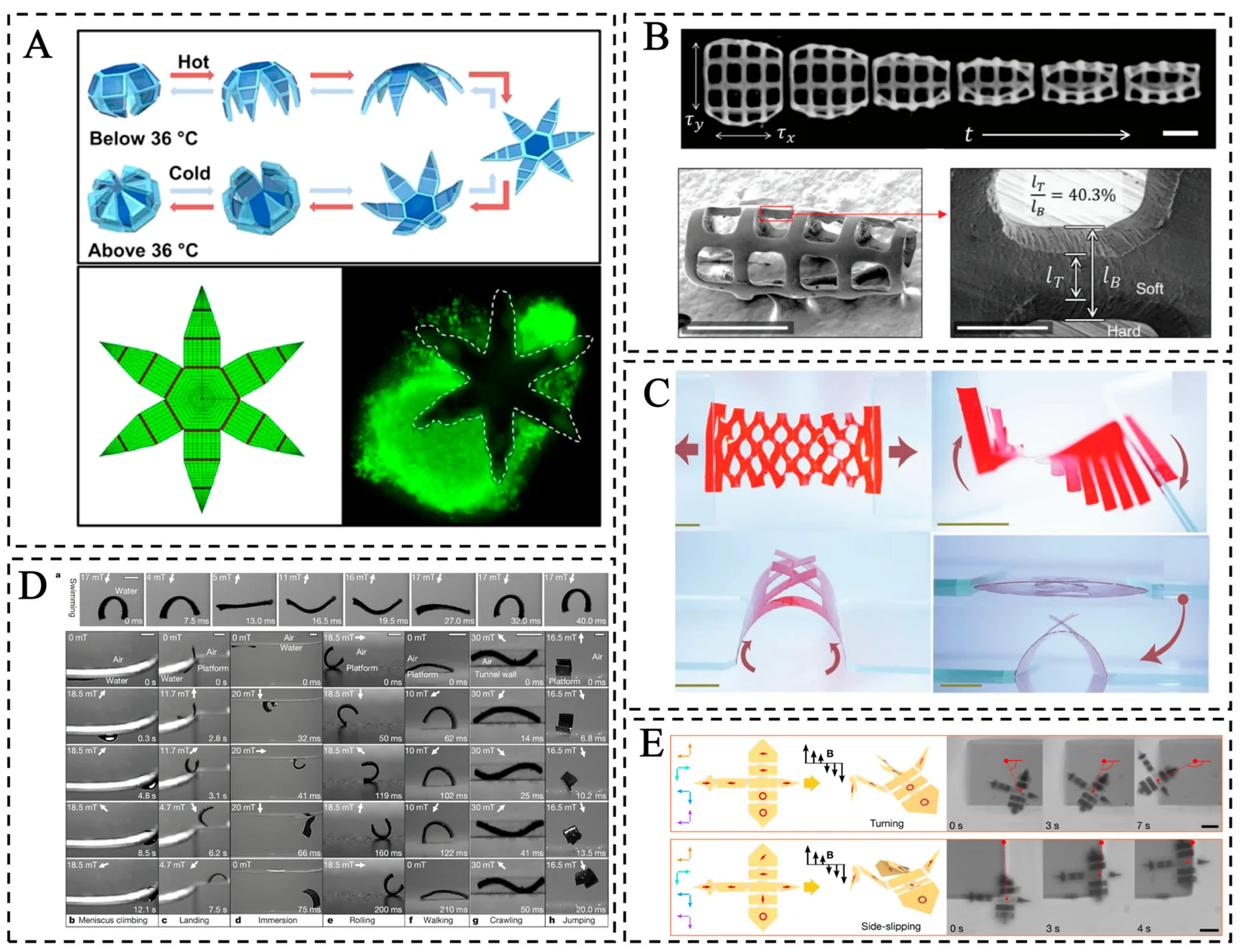
The soft microrobots. (**A**) The bilayer thermo-magnetically responsive soft microgrippers (reprinted from Ref. [115] Copyright © 2015, American Chemical Society). (**B**) The single-layer self-rolled microrobot (reprinted from Ref. [125] Copyright © 2021, Wiley-VCH GmbH). (**C**) The Kirigami-based light-induced shape-morphing microrobots (reprinted from Ref. [129] Copyright © 2020, Wiley-VCH GmbH). (**D**) The soft-bodied microrobots with multimodal locomotion (reprinted from Ref. [134] Copyright © 2018, Macmillan Publishers Limited). (**E**) The nanomagnetic encoding of shape-morphing microrobots (reprinted from Ref. [138] Copyright © 2019, Springer Nature Limited).

Bilayer/multilayer beam deformation structures are a typical configuration of predefined deformation structures [115,116,117,118,119,120,121,122,123]. Their structural characteristic is the superposition of two or more layers of materials with different stimuli-responsive properties along the thickness direction. By compositing material layers with different thermal expansion coefficients, swelling ratios, or response characteristics, bending deformation is achieved through interlayer strain mismatch. For instance, Breger et al. designed a finger-like bilayer microgripper in which polypropylene fumarate (PPF) segments are patterned atop a continuous poly (N-isopropylacrylamide-co-acrylic acid) (pNIPPAm-AAc) hydrogel layer. The PPF segments serve as geometric constraints that localize differential swelling-induced bending to discrete inter-segmental gaps, enabling the planar sheet to reversibly fold into a three-dimensional gripper capable of grasping and releasing (Figure 3A) [115]. Hippler et al. demonstrated that spatially varying the laser exposure dose during two-photon lithography locally tuned the crosslinking density of poly (N-isopropylacrylamide) (pNIPAAm) hydrogel within a single resist, enabling complex three-dimensional architectures with large-amplitude, programmable deformation that could be spatially controlled via localized two-photon heating [116]. Wang et al. developed an asymmetric polypyrrole/polyethylene glycol terephthalate (PPy/PET) bilayer actuator in which a hierarchically micro/nanostructured PPy layer stacked on a PET substrate generated intrinsic differential deformation in response to humidity, light, electric, and thermal stimuli. Laser-carved geometric patterns in the PPy film dictated reversible transitions between flat 2D sheets and curved 3D architectures, while the spiral-shaped bilayer geometry enabled temperature-gradient-driven ultrafast rolling and light-induced crawling locomotion [117]. In biomedical applications, the temperature-sensitive and pH-responsive properties of these structures hold broad application prospects for drug release triggered by body temperature or microenvironment changes, as well as for tissue engineering scaffolds [118].

Single-layer beam structures achieve deformation through asymmetric geometry or asymmetric stimulus loading of a single material [124,125,126,127,128]. Unlike bilayer/multilayer structures, their deformation does not rely on differential expansion at material interfaces. Instead, it arises from structural geometric asymmetry (e.g., one end fixed, one end free) or from the spatially non-uniform distribution of an external stimulus (e.g., local heating by focused light) to produce bending deformation. For example, Ye et al. developed a single-layer hydrogel microrobot in which voxel-programmed intrinsic stress gradients combined with integrated hinge designs enabled rapid 2D-to-3D shape transformation within seconds, achieving multifunctional capabilities such as targeted drug release, cargo transport, and modular assembly without requiring multi-material architectures [124]. Nguyen et al. fabricated a single-layer microrobot in which anisotropic photoprinted planar patterns introduced crosslinking density gradients, driving spontaneous self-rolling of the structure into a hollow cylindrical configuration. The resulting tubular architecture enabled magnetic targeting, co-loading of contrast agents and therapeutic drugs, near-infrared-triggered localized drug release, and complete retrievability (Figure 3B) [125]. The advantage of such structures lies in their structural simplicity and rapid response, but their deformation modes are relatively limited compared to multilayer configurations.

Origami/kirigami structures achieve controllable transformation from two-dimensional (2D) planes to 3D complex configurations under external stimuli by introducing predefined fold lines (origami) or cut patterns (kirigami) into 2D thin films or sheet materials, thereby encoding macroscopic deformation degrees of freedom into localized hinge motions [129,130,131,132,133]. Unlike single-layer and bilayer beams, which rely on the intrinsic expansion or contraction of the material itself to generate deformation, origami/kirigami structures derive their deformation from the concentrated motion of geometrically predefined weakened regions (fold lines or cuts) under stress, while the rigid panel regions undergo negligible deformation. For instance, Chen et al. developed a kirigami-based light-fueled robotic structure, in which external stress fields induced out-of-plane deformation of liquid crystal network films, enabling 2D-to-3D shape transformations and mechanical actuation upon light illumination (Figure 3C) [129]. Novelino et al. developed a magnetically actuated origami system based on bistable Kresling patterns, in which distributed magnetic plates attached to individual unit cells generate localized torques that drive untethered, rapid shape transitions with instantaneous state locking. This segmented spatial architecture enables independent control of each cell and allows for on-demand multimodal structural reconfiguration [130]. The advantage of origami/kirigami structures lies in their high determinacy and repeatability in deformation.

Predefined deformation structures, whose deformation pathways are fixed during fabrication, face inherent limitations when operated within the dynamic and heterogeneous milieus of biomedical applications. In vivo conditions, including vascular geometry, tissue stiffness, and local biochemical gradients, differ substantially across patients and even within the same individual over time. A structure designed to undergo a specific bending or folding sequence under a presumed stimulus may fail to deform appropriately when the actual physiological environment deviates from the design assumptions, leading to compromised navigation, inaccurate payload release, or unintended tissue interaction. This rigidity in behavioral programming thus motivates the development of dynamically reconfigurable structures, which can adjust their deformation mode in real time according to the encountered conditions, offering a more robust and adaptive solution for biomedical microrobots.

### 4.2. Dynamically Reconfigurable Structures

Dynamically reconfigurable structures enable online switching of deformation modes by adjusting the parameters of the external actuation field or modulating the spatial distribution of responsive materials embedded within the structure. The physical basis for this capability lies in the fact that the deformation forces originate from the interaction between the external field and responsive components. Based on the physical mechanism underlying deformation reconfiguration, these structures can be categorized into three types, namely external field parameter-driven reconfigurable structures, internal heterogeneity programming reconfigurable structures, and hybrid-driven reconfigurable structures.

External field parameter-driven reconfigurable structures are composed of relatively homogeneous stimuli-responsive materials without complex spatially encoded heterogeneity within the structure [134,135,136,137]. The switching of deformation modes relies entirely on real-time variations in the parameters of the external actuation field, such as frequency, amplitude, direction, and wavelength. Different deformation modes arise from differences in the global dynamic response of the structure induced by changes in the external field. For example, Hu et al. developed rectangular sheet-shaped soft microrobots with spatially encoded harmonic magnetization geometry throughout the elastomer matrix. The ordered arrangement of NdFeB microparticles generates a differential torque distribution under magnetic stimulation, driving reversible reshaping of the planar sheet into curved 3D profiles. These geometry-derived morphological transformations enable diverse locomotion modes across multiple terrains (Figure 3D) [134]. Yang et al. demonstrated that one-dimensional colloidal chains with multiblock asymmetry can fold into complex geometries including helices, plectonemes, lassos, and coils by varying the applied magnetic field [135]. The advantage of those structures lies in their relatively simple fabrication process, which does not rely on complex internal encoding procedures.

Internal heterogeneity programming reconfigurable structures embed spatial inhomogeneities, such as magnetization direction distributions, into the material during the fabrication stage [138,139,140,141]. Under a uniform or slowly varying external field, these pre-encoded heterogeneities trigger differentiated local responses that drive complex three-dimensional deformations, with the deformation mode switchable online by varying the amplitude or direction of the external field. For instance, Cui et al. encoded multiple shape-morphing instructions into a micromachine by programming single-domain nanomagnet arrays on connected panels via tailored magnetic field sequences, demonstrating modular units that morphed into alphabet letters and a microscale bird capable of flapping, hovering, turning, and side-slipping (Figure 3E) [138]. Lahikainen et al. developed a single light-active actuator capable of achieving six distinct shape reconfigurations under identical illumination conditions by synergistically combining photochemical azobenzene isomerization with photothermal effects in liquid crystal polymer networks [139]. This design strategy extends structural programming from macroscopic geometric design to the regulation of internal material structure at the magnetic domain scale, vastly expanding the design space while enabling rich and precise shape reconfiguration under simple uniform external fields.

Hybrid-driven reconfigurable structures integrate real-time modulation of external field parameters with internal heterogeneity encoded during the fabrication stage. For example, Huang et al. constructed composite microswimmers featuring partitioned bilayer and monolithic hydrogel geometries, in which pre-oriented magnetic nanoparticle arrays encode tunable folding behavior, and the patterned body flagellum architectures enable multiple switchable swimming modalities under combined magnetic and thermal control [142]. Those structures substantially expand the dimensionality and complexity of achievable deformation modes by simultaneously exploiting the differentiated response characteristics pre-encoded within the material and variations in external actuation field parameters.

### 4.3. Advantages and Limitations of Soft Structures

The main advantage of soft microrobots lies in the enhancement in biomechanical compatibility. Low-modulus materials are expected to reduce the contact stress with biological tissues, minimizing mechanical damage risks faced by rigid structures [30]. Deformable structures may pass through narrow anatomical spaces that rigid microrobots cannot navigate, offering advantages in complex geometries such as vascular bifurcations. Furthermore, the diversification of motion modalities opens possibilities for multi-functional integration.

While soft architectures endow microrobots with high mechanical compliance and biosafe environmental adaptability, they often exhibit reduced force output and motion precision under conventional operational conditions, both of which are essential for many biomedical interventions. Soft microrobots typically generate nanonewton-level forces, insufficient to penetrate dense extracellular matrices or perform mechanical work on stiff tissues [143]. Moreover, the theoretically infinite degrees of freedom in continuum deformation of soft structures complicate precise motion prediction and control [134]. Essentially, this inherent contradiction among structural compliance, force output capability, and motion controllability constitutes a central challenge in soft microrobots, as pure soft systems find it difficult to simultaneously achieve safe biological interaction and high-force functional actuation [144]. However, this trade-off is not universal, as it can be modulated by architecture, material reinforcement, actuation mechanism, length scale, and operating environment. Accordingly, to overcome this limitation and integrate the complementary advantages of soft adaptability and high-performance force output, rigid–soft integrated microstructures have emerged as a feasible direction for advanced biomedical microrobotic systems.

## 5. Rigid–Soft Integrated Microrobots

Rigid structures are generally precise and forceful but lack compliance, whereas soft structures offer safety and compliance but limited force output. Rigid–soft integrated structures have been proposed to combine the advantages of both approaches, aiming to achieve task-dependent synergy between the actuation force and control precision of rigid components and the environmental adaptation capabilities of soft components. However, such synergy is not automatic but must be engineered through deliberate spatial or temporal partitioning, and it requires experimental validation against appropriate pure-rigid and pure-soft benchmarks. From an engineering perspective, the physical realization of rigid–soft integration can be achieved through two fundamentally distinct design pathways. The first is permanent spatial heterogeneity, in which rigid and soft components are distributed in distinct regions through material arrangement during fabrication. The second is genuine on-demand variable stiffness, in which the same material or structure operates at different stiffness levels depending on the task requirement or environmental stimulus. The first approach achieves functional partitioning through spatial material and structure arrangement during the fabrication process, whereas the second enables the system to exhibit different stiffness characteristics at different times, being rigid during manipulation phases and soft during adaptation phases. These two strategies differ substantially in mechanism, reversibility, energy demand, switching time, fatigue behavior, achievable stiffness range, and biocompatibility.

### 5.1. Spatially Heterogeneous Integration Structures

Spatially heterogeneous integration structures distribute rigid and soft components at discrete physical locations within the microrobot, a design strategy inspired by biological systems in which rigid bones and soft muscles operate synergistically to achieve both structural support and adaptive motion. Based on the spatial organization of rigid and soft components, they can be subdivided into segmented joint configurations, origami/kirigami-inspired designs, and rigid-frame/soft-shell architectures.

**Figure 4:**
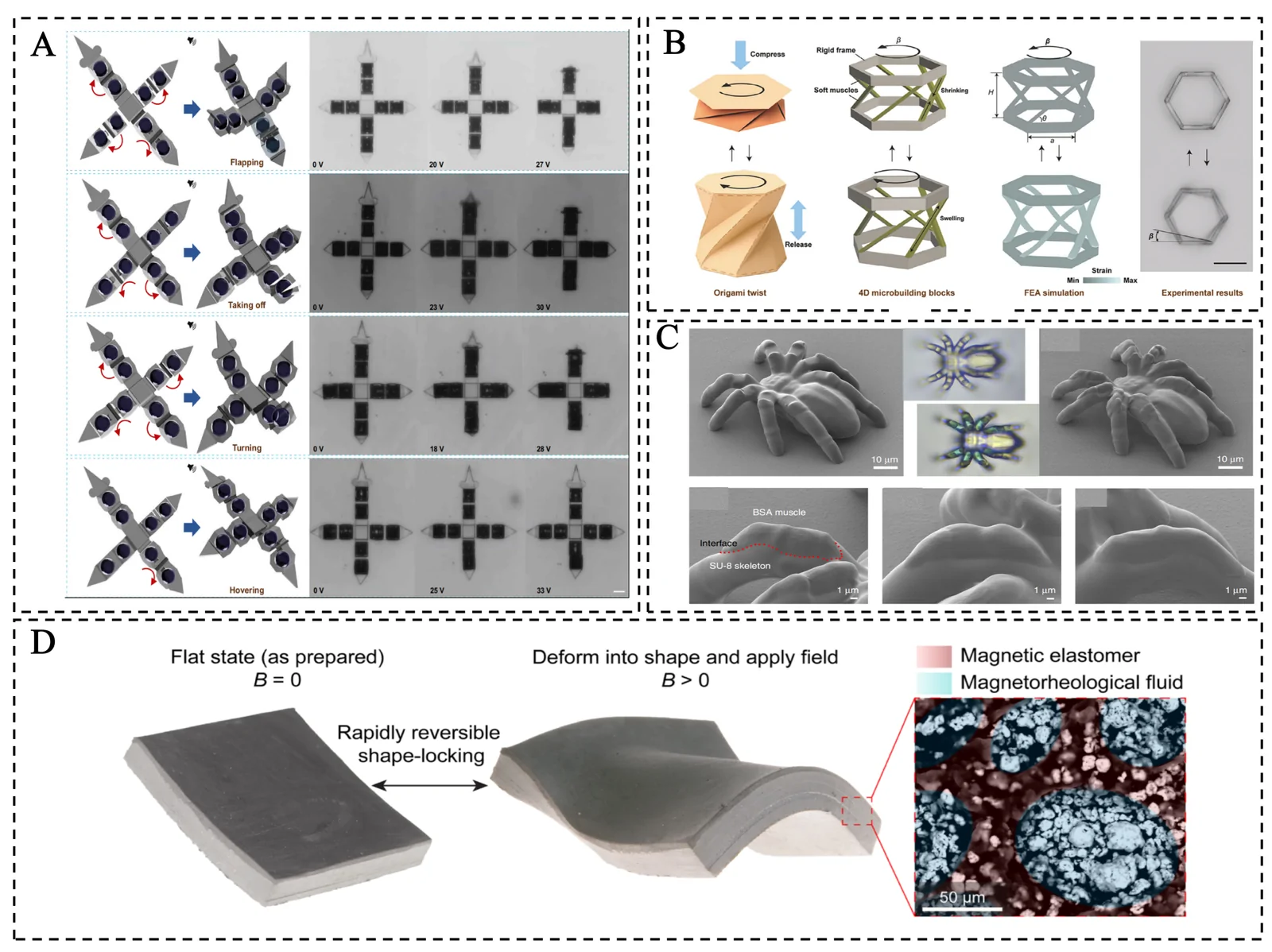
The rigid–soft integrated microrobots. (**A**) The acoustic shape-morphing microrobots (reprinted from Ref. [147] Copyright © 2026, Springer Nature Limited). (**B**) The origami twist-inspired shape-morphing microrobots (reprinted from Ref. [153] Copyright © 2026, Wiley-VCH GmbH). (**C**) The programmed artificial musculoskeletal microrobots (reprinted from Ref. [157] Copyright © 2020, Springer Nature Limited). (**D**) The magnetorheological elastomers for rapid and extreme stiffness tuning (reprinted from Ref. [162] Copyright © 2023, Royal Society of Chemistry).

Segmented joint configurations arrange rigid segments and soft segments alternately or sequentially along the length of the microrobot, representing a direct approach to spatially heterogeneous integration [147,148,149,150,151]. Rigid segments, typically composed of high-modulus materials or high-stiffness geometric configurations, provide force transmission and structural support, while soft segments, composed of low-modulus elastomers or hydrogels, provide compliance and deformation degrees of freedom. For instance, Su et al. developed an acoustic micromachine composed of two rigid microbubble frames connected by a soft microhinge that achieved millisecond-scale deformation through acoustic-field-induced bubble interactions, enabling programmable multi-mode switching and precise control via tunable excitation amplitude (Figure 4A) [147]. Watanabe et al. developed a soft-rigid hybrid microrobot composed of pNIPAAm and a rigid photoresist, in which graphene incorporated into the pNIPAAm matrix imparted high photothermal conversion efficiency, enabling sequential actuation through localized heating via focused light irradiation [148]. The advantage of such structures lies in their design simplicity and the functional independence of each module. However, mechanical reliability at inter-segment interfaces represents a critical challenge, as stress concentrations on rigid/soft interfaces may easily cause failure of segmented microswimmers during long-term operation.

Origami/kirigami-inspired rigid–soft integrated structures employ rigid panels to provide structural support and force transmission pathways, and soft hinges to provide deformation degrees of freedom [152,153,154,155,156]. In those configurations, panels remain largely undeformed during structural actuation, with mechanical energy primarily stored in the crease or linkage regions. This design paradigm enables predictable kinematic behavior while allowing for programmable shape morphing through controlled folding and cutting patterns. For instance, Zhu et al. developed an electrothermal micro-origami system that employed localized Joule heating for rapid elastic folding and overheating for plastic folding to reprogram static geometry, thereby enabling environment-independent multi-degree-of-freedom shape morphing and complex motions [152]. Wang et al. developed origami-inspired rigid–soft coupled microbuilding blocks by integrating high-stiffness frames with soft hydrogel actuators, achieving high torsional deformation through parametric optimization, with modular assembly into chain structures further enabling complex three-dimensional shape morphing and magnetic locomotion via selective magnetic material integration (Figure 4B) [153]. From a mechanical essence perspective, origami/kirigami structures leverage patterning design during the fabrication stage to achieve modulus distribution control in space.

Rigid-frame/soft-shell structures feature an internally rigid framework that provides mechanical support and actuation, and an externally soft shell that mediates environmental interaction, reduces friction, and minimizes mechanical damage [157,158,159,160,161]. This clear functional division between rigid and soft components ensures both actuation efficiency and structural integrity while potentially improving biocompatibility at the biological interface, though comprehensive in vivo validation remains necessary. For example, Ma et al. developed femtosecond laser-programmed artificial musculoskeletal microbots featuring stiff SU-8 skeletons and soft pH-responsive bovine serum albumin muscles, as demonstrated by a spider microbot and a smart microgripper capable of controllable grabbing and releasing (Figure 4C) [157]. Tang et al. designed a biodegradable magnetic microrobot with a core–shell architecture for the co-delivery of stem cells and bioactive molecules, in which a biodegradable magnetic microsphere core enabled external magnetic navigation and sustained drug release while a mesenchymal stem cell outer shell ensured therapeutic viability [158]. This design paradigm holds application prospects for implantable medical devices requiring simultaneous satisfaction of structural strength requirements and tissue safety standards.

### 5.2. Temporal Stiffness Modulation Structures

In contrast to permanent spatial partitioning, temporal stiffness modulation structures enable the same material or structure to exist in different stiffness states at different points in time, allowing for on-demand stiffness adjustment that approximates the adaptive behavior of biological tissues. This paradigm can be realized through two fundamentally distinct approaches. Material-based strategies reversibly alter the intrinsic elastic modulus of the constituent material in response to external stimuli. Structure-based strategies modulate the effective structural stiffness through geometric reconfiguration, folding, or variable mechanical linkage while keeping the material modulus constant. These two strategies differ fundamentally in their physical mechanisms, reversibility, energy demand, switching kinetics, fatigue behavior, achievable stiffness range, and biocompatibility. Meanwhile, it must be acknowledged at the outset that genuine temporal stiffness modulation at the microscale remains exceptionally rare. The overwhelming majority of existing variable-stiffness platforms have been demonstrated either as bulk material specimens or as millimeter-scale devices, and their direct translation into autonomous biomedical microrobots faces formidable physical barriers.

Material-based stiffness modulation strategies achieve temporal stiffness modulation by reversibly altering the intrinsic elastic modulus of constituent materials. In these systems, external stimuli induce physical or chemical transitions, such as crosslink density variations, polymer hydration state switches, or phase changes, which directly alter the intrinsic deformation resistance of the material at the molecular scale [162,163,164,165,166,167,168,169,170,171,172]. Representative material systems encompass viscosity-tunable fluids, including magnetorheological and electrorheological variants, as well as phase-change materials such as shape-memory polymers and low-melting-point alloys. However, current research on material-based stiffness modulation structures has primarily focused on the material level and the millimeter scale, while studies at the sub-micron scale remain scarce. For instance, Barron et al. proposed a design strategy for magnetorheological elastomers (MREs) by embedding a combination of magnetic particles and ferrofluid into an elastomer matrix, achieving a stiffness modulation ratio of up to 70-fold with a response time of approximately 20 ms and reversible shape-changing capability (Figure 4D) [162]. Schubert et al. developed a variable-stiffness material by embedding a rigid low-melting-point alloy microstructure within a soft poly (dimethylsiloxane) (PDMS) matrix, achieving a stiffness change exceeding 25-fold with rapid switching from rigid to soft states in under one second at low power consumption [163]. Min et al. developed a magnetorheological elastomer-based millirobot whose elastic modulus can be switched from 0.477 to 2.03 MPa under an external magnetic field, enabling rapid, reversible stiffness modulation [164]. The robot demonstrated controllable adhesive capability for gripping, lifting, and transporting delicate biological tissues.

These examples are better classified as material-level enabling technologies rather than microscale robotic implementations. Their direct downscaling is impeded by diffusion-limited kinetics that constrain the stiffness modulation range at small scales, by inter-particle distances in microscale magnetorheological composites that approach particle diameters and hinder field-induced chain formation, by prohibitive thermal management because heat dissipation scales unfavorably with size, and by cumulative cycling damage such as network fatigue, particle aggregation, and phase separation, whose fatigue life under physiological conditions remains uncharacterized. Furthermore, leakage of functional components, including magnetic nanoparticles, liquid metal droplets, or unreacted crosslinkers, into surrounding tissues poses a severe biocompatibility hazard. Consequently, genuine material-based temporal stiffness modulation has not yet been demonstrated in a microrobot under physiological conditions.

Structure-based stiffness modulation strategies realize temporal stiffness modulation through dynamic geometric reconfiguration, including adjustment of component overlap ratios, linkage engagement, or folding states, to modify load-dependent effective structural stiffness without altering the intrinsic elastic modulus of constituent materials [173,174]. For example, Liu et al. developed a magnetically controlled helix-shaped microrobot (Helixoft) that integrates with commercial microcatheters down to 300 μm in diameter. By dynamically rotating a rigid magnetic helix to adjust its overlap with a soft microtube, the system achieves 40-fold stiffness tuning and active steering of up to 118°, with validation demonstrated for deep-bronchial delivery and oviduct biopsy in live pigs [173]. Nevertheless, it should also be noted that this example is more appropriately classified as a conceptual-level enabling technology rather than a functional microrobot. Compared with material-based counterparts, structure-driven temporal stiffness tuning avoids material-level phase transitions and chemical bond rearrangement, thereby circumventing, in principle, risks such as phase segregation, leakage of material phases, and thermally induced tissue damage.

### 5.3. Advantages and Limitations of Rigid–Soft Integrated Structures

Rigid–soft integrated structures, through the spatiotemporal programmability of their mechanical properties, show considerable potential to reconcile the competing demands of compliant navigation and forceful manipulation within a single microrobotic system. Whether such integration genuinely outperforms pure rigid or pure soft counterparts depends on the specific task, architecture, and benchmarking criteria. Nevertheless, three principal advances characterize this paradigm. First, performance synergy rather than compromise, which overcomes the traditional dichotomy that rigidity precludes compliance and vice versa. Second, expansion of functional dimensionality, whereby spatial rigid–soft partitioning enables independent optimization of distinct body regions and temporal stiffness switching allows a single region to adapt to varying mechanical requirements across mission phases. Third, enhanced biomechanical compatibility, in which soft encapsulating shells or switchable stiffness mechanisms may mitigate mechanical mismatch and interfacial stress at the tissue interface without compromising functionality, although their long-term effects on tissue remodeling and inflammatory responses warrant further investigation.

Despite these advances, rigid–soft integrated structures remain at a nascent stage confronted with multiple intertwined engineering and biological bottlenecks, whereby spatially heterogeneous and temporally stiffness-modulated architectures exhibit distinct failure modes. For permanent spatially heterogeneous microrobots, heterogeneous material interfaces represent the primary vulnerability, as repeated cyclic deformation under physiological fluid loading can induce interfacial fatigue, debonding, and fragment shedding. Released micro-debris within biofluids may trigger thrombosis or immune-mediated foreign-body responses. Meanwhile, temporally stiffness-modulated systems suffer from additional failure pathways. Material-based variable-stiffness designs are susceptible to mechanical hysteresis and leakage or migration of functional components upon repeated stimulus cycling, while stimulus-associated side effects including local thermal exposure from phase-change actuation and high-magnitude magnetic field requirements impede in vivo translation. For structure-based stiffness modulation concepts, cyclic operation generates frictional wear and contact-driven hysteresis at movable joints and contact interfaces. Notably, most temporally modulated demonstrators exist only as material-level specimens, millimeter-scale prototypes, or tethered enabling technologies, while fully validated untethered microscale biomedical microrobots remain scarce.

Three universal bottlenecks affect all rigid–soft integrated architectures. First, manufacturing complexity stems from multi-material microfabrication, embedding of functional inclusions, and precision assembly of micro-linkages, which impose substantial technical barriers, while scalable low-cost batch-manufacturing pipelines remain immature. Second, modeling and control challenges arise from the absence of well-established theoretical frameworks for multi-physics, multi-timescale, and multi-spatial-scale coupled dynamics, which restricts the development of reliable closed-loop control for complex physiological environments. Third, inherent safety hazards arise because interfacial debonding may release payloads or expose rigid inner cores, and abrasive wear can produce microscale debris that carries embolization risks. Collectively, structural limitations, fabrication barriers, control difficulties, and safety concerns constitute the major bottlenecks constraining further progress of rigid–soft integrated biomedical microrobots.

To quantitatively reveal the intrinsic trade-offs among precision, output force, compliance, and environmental adaptability across rigid, soft, and rigid–soft integrated microrobots, Table 1 summarizes key performance and experimental parameters of representative systems. Critical quantitative indices include geometric dimension, material composition, effective elastic modulus or stiffness, actuation principle, locomotion behavior, speed, output force, deformation or stiffness-tuning range, response time, payload capacity, fabrication route, biological test model, biodegradability, imaging and tracking modality, and experimental validation level.

## 6. Clinical Translation of Microrobots

### 6.1. Biomedical Applications and Recent Progress

Despite successful demonstrations of these structural paradigms in diverse settings, their clinical translation demands rigorous systematic assessment. To date, the most extensively pursued application remains targeted drug delivery, in which microrobots serve as active carriers capable of transporting therapeutic agents against passive diffusion and elevated interstitial fluid pressure [1,2]. Recent clinically oriented magnetic microrobot platforms, which integrate dissolvable drug-loaded capsules with electromagnetic navigation, have demonstrated targeted therapy in human vasculature models and in large-animal trials involving sheep and pigs under realistic clinical conditions [175]. In respiratory therapy, inhalable biohybrid microrobots have emerged as a non-invasive strategy for pulmonary treatment, demonstrating active nebulization-based delivery and therapeutic efficacy in a mouse model of acute methicillin-resistant *Staphylococcus aureus* pneumonia with pulmonary retention exceeding five days [176]. Likewise, biohybrid microrobots functionalized with drug-loaded nanoparticles have been shown to inhibit the progression of lung metastasis in murine models following intratracheal administration [177]. In imaging-guided therapy, early studies have also demonstrated controlled in vivo swimming of bacteria-like microrobotic flagella in living animals [76], as well as photoacoustic computed tomography-guided targeted navigation in intestines in vivo [178]. Multifunctional biohybrid magnetite microrobots have further validated imaging-guided therapeutic capabilities in biological environments [179]. In addition, multifunctional microrobots with real-time MRI visualization have been employed for chemoembolization therapy of liver cancer in vivo [180]. In urinary tract therapy, imaging-guided bioresorbable acoustic hydrogel microrobots have demonstrated effective tumor shrinkage in mouse bladder tumor models over a 21-day treatment course [181]. In addition, surface microrollers have demonstrated targeted cargo delivery under physiological blood flow conditions [182].

### 6.2. In Vivo Validation and Physiological Barriers

The clinical translation of microrobots is typically assessed through a four-tier validation hierarchy comprising proof-of-concept locomotion in idealized media, in vitro biological validation, ex vivo testing in explanted tissues or organs, and in vivo demonstration in living organisms. Most reported microrobots have only achieved the first two tiers and limited ex vivo testing, while comprehensive benchmarking across all four tiers remains rare [14,15,16,17,18,19,20,21,22,176,177,178,179,180,181,182]. A critical gap in the current literature is the frequent extrapolation from proof-of-concept locomotion in deionized water, hydrogen peroxide solutions, or patterned substrates to presumed functionality in physiological environments, which can lead to significant performance deviations when these microrobots are deployed in actual clinical applications.

Each structural class faces distinct physiological barriers. Rigid microrobots operating in blood encounter viscosities substantially higher than water, which drastically alters propulsion efficiency and magnetic torque requirements. Protein adsorption and the subsequent formation of a protein corona modify surface properties and can abrogate the asymmetric catalytic activity of Janus motors [102]. Furthermore, their inability to deform in response to vessel diameter variations poses a significant risk of embolization in narrow capillaries. Soft microrobots, although mechanically better matched to tissues, suffer from reduced propulsion force in high-viscosity media and may undergo uncontrolled swelling or degradation in ionic physiological fluids, which complicates motion prediction. Rigid–soft integrated systems must also withstand pulsatile flow and cyclic mechanical loading at heterogeneous interfaces, where delamination or fatigue failure could release fragmented components into the bloodstream [158]. Table 2 presents a SWOT (Strengths, Weaknesses, Opportunities, Threats) analysis for each structural paradigm with respect to clinical translation.

Table 2 reveals that no single structural paradigm is unconditionally superior for clinical translation. Rigid systems excel in controlled mechanical output but pose acute safety risks in soft-tissue environments. Soft systems maximize biological compatibility yet lack the force and precision required for active therapeutic intervention. Rigid–soft integrated architectures theoretically resolve this conflict, but they introduce new vulnerabilities at material interfaces that have not been experimentally characterized over clinically relevant timescales. The selection of a structural paradigm must therefore be dictated by the specific anatomical target, therapeutic payload, and the mechanical interaction required, rather than by universal performance metrics.

### 6.3. Safety and Clinical Risk Assessment

It should be noted that although current studies have demonstrated the safety of microrobot materials or structures in vitro, deviations may arise in actual clinical applications. In fact, biocompatibility is context-dependent and provisional, and safety conclusions based solely on material selection or structural compliance cannot be fully validated in clinical settings. Safety is an emergent property governed by material composition, dosage, degradation products, field exposure, and clearance kinetics, and must therefore be evaluated from three interrelated perspectives. First, material and chemical safety extends beyond bulk biocompatibility to leachable toxins and the unpredictability of degradation. Nickel-based components can release cytotoxic Ni^2+^ upon corrosion, while hydrogen peroxide-fueled tubular motors generate oxidative stress incompatible with clinical use. Biodegradable polymers may undergo premature hydrolysis in the presence of tissue esterases, whereas non-degradable metals or SU-8 photoresists risk indefinite retention unless reliable retrieval is guaranteed. Second, biological interfacial safety concerns the microrobot-tissue boundary. Protein corona formation can mask surface functionalization and trigger immune recognition, leading to macrophage engulfment and fibrotic encapsulation within days [183]. For rigid structures in the vasculature, stagnation or endothelial irritation may initiate thrombosis and embolization [184]. Soft hydrogels reduce contact stress, yet excessive deformation in narrow capillaries can occlude microvessels and compromise perfusion. Third, physiological barriers and systemic safety encompass clearance, navigation, and chronic exposure. Dense extracellular matrices and viscoelastic mucus layers in the gastrointestinal and respiratory tracts can immobilize particles indefinitely [176,185], while renal and hepatic clearance thresholds impose strict constraints on size and surface chemistry. Magnetic retrieval becomes unreliable if demagnetization or fragmentation occurs, and prolonged exposure to alternating magnetic fields may induce eddy-current heating or neurological perturbations. In conclusion, while current research has made significant contributions to the clinical translation of microrobots, this represents only the initial stage of that process [186]. Consequently, safety conclusions derived from existing studies cannot be directly extrapolated to actual clinical applications.

## 7. Conclusions and Outlook

### 7.1. Conclusions

This review has systematically examined the structural evolution of biomedical microrobots, tracing the developmental trajectory from rigid architectures through soft configurations to rigid–soft integrated systems. This evolutionary progression reflects the continuous refinement of structural design strategies in response to the increasingly demanding requirements of biomedical applications. Rigid microrobots, constructed from high-modulus materials and relying on geometric or surface asymmetry, have demonstrated precise and controllable locomotion in low-Reynolds-number environments. Supported by well-established theoretical frameworks and fabrication protocols, they have been successfully applied in targeted delivery and cell manipulation. However, their inherent structural non-compliance fundamentally limits their adaptability to complex physiological environments and introduces risks of mechanical tissue trauma. Soft microrobots circumvent the inherent constraints of rigid counterparts by exploiting low-modulus materials that afford programmable shape-morphing capabilities. Through the deliberate encoding of deformation trajectories into both compositional gradients and internal structural hierarchies, these systems achieve exceptional biomechanical compatibility and adaptive responsiveness to diverse operational environments. Predefined deformation architectures enable deterministic and stimulus-triggered shape transformations, whereas dynamically reconfigurable designs extend functional versatility by allowing for on-the-fly switching between distinct deformation regimes via external field modulation and internal material distribution. However, a persistent and fundamental trade-off persists between mechanical compliance and actuation output. The very low elastic modulus that underpins environmental adaptability inevitably curtails force generation capacity and diminishes motion fidelity, posing a critical bottleneck for practical applications. Rigid–soft integrated structures constitute the current frontier in microrobot structural design, as they aim to reconcile the inherent conflict between compliant navigation and forceful manipulation within a unified framework. Spatially heterogeneous integration enables the strategic distribution of rigid and stiff components to achieve functional partitioning, while temporal stiffness modulation affords on-demand transitions between compliant and rigid states. However, demonstrating their comprehensive performance superiority conclusively requires head-to-head benchmarking against equivalent rigid and soft microrobot counterparts, and several practical engineering bottlenecks remain unresolved in existing prototypes. Collectively, these strategies have preliminarily demonstrated the feasibility of transcending the conventional rigidity-compliance dichotomy.

### 7.2. Outlook

In addition to the structural advances surveyed above, the integration of artificial intelligence and intelligent control systems is reshaping microrobot capabilities. Machine learning algorithms, particularly deep learning and physics-informed neural networks, have been employed to predict locomotion dynamics, optimize structural geometries, and accelerate design iterations beyond the reach of conventional methods [187]. Reinforcement learning frameworks enable model-free closed-loop control, allowing magnetic microrobots to autonomously navigate fluidic environments and adapt to unmodeled perturbations without prior dynamic system identification [188,189]. Concurrently, swarm intelligence strategies leveraging heterogeneous collectives have demonstrated emergent behaviors such as collective object caging and adaptive pattern formation, thereby expanding functional repertoires beyond the limitations of individual agents [190,191]. Deep learning-based orientation estimation and photoacoustics-guided closed-loop systems have achieved real-time tracking and trajectory correction under dynamic physiological disturbances [192,193]. Digital twin platforms, which are virtual replicas synchronized with physical microrobot states, further bridge the simulation-to-clinic gap by enabling preoperative planning and intraoperative monitoring with near-millisecond latency [194]. Collectively, these intelligent methodologies address the critical bottleneck of operating in unpredictable biological environments, although the reality gap between idealized training conditions and complex physiological milieus remains a key challenge.

Looking forward, several research directions hold particular promise for advancing the structural design of biomedical microrobots.

First, the emergence of data-driven structural optimization frameworks that integrate machine learning with multi-physics modeling represents a burgeoning paradigm [187,195], which, though still in its early stages, holds considerable potential for accelerating the discovery of optimal configurations for specific biomedical tasks. However, the direct use of these methodologies for microrobot structural design requires the establishment of open-access locomotion performance databases, standardized multi-physics validation workflows, and experimentally benchmarked performance metrics.

Second, reconfigurable architectures capable of autonomous adaptation to unpredictable physiological microenvironments remain a central challenge. Current programmable structures rely primarily on fabrication-stage encoding that cannot be modified after deployment. Material-based and structure-based stiffness modulation microrobots represent promising solutions to this challenge. Future progress therefore depends not only on the discovery of new materials, but also on advances in microscale manufacturing technologies. Equally critical, experimental benchmarking protocols must quantify reconfiguration speed, long-term cycling stability targeting millions rather than hundreds of cycles, energy cost, and functional reliability under physiologically realistic conditions that encompass protein adsorption, ionic strength fluctuations, and pulsatile flow.

Third, multifunctional hybrid materials represent a critical frontier for expanding the functional repertoire of microrobots, for instance, the integration of therapeutic, biosensing, and imaging functionalities within unified microscale architectures. Future structures can spatially orchestrate functional polymers, magnetic nanocomposites, and bioactive hydrogels to create theranostic platforms capable of simultaneous diagnosis and intervention. However, the structural integration of such disparate material components not only demands advanced microfabrication protocols but also requires overcoming substantial challenges in interfacial compatibility and stress transfer efficiency.

Fourth, wireless energy transfer strategies must be integrated directly into the microrobot structure to overcome the penetration limits of externally applied fields. Embedding resonant electromagnetic microstructures or ultrasound-responsive cavities within the microrobot body can enable localized energy harvesting, yet substantial challenges remain in optimizing transfer efficiency while maintaining biocompatible power densities for deep-tissue operation.

Fifth, self-powered microrobots offer an alternative paradigm that eliminates reliance on external power sources by harvesting ambient or biochemical energy from the surrounding environment. The convergence of structural design with emerging energy-harvesting materials, including piezoelectric polymers that exploit mechanical deformation to generate charge and biofuel cells that draw energy from endogenous glucose or lactate, could ultimately enable autonomous, battery-free microrobots. However, architectural innovations must seamlessly couple energy conversion, storage, and actuation modules within single microscale frameworks, which requires experimental validation of power output against locomotion energy budgets.

Sixth, structural intelligence, one of the ultimate goals of structural evolution, aims to enable the mechanical structure itself to achieve sensing, decision-making, and execution functions through embedded nonlinear mechanical responses [196,197]. This paradigm moves beyond passive shape morphing toward active and autonomous behavior, enabling untethered microrobots to execute adaptive tasks in dynamic physiological niches. Achieving structural intelligence at the micrometer scale requires the development of design methodologies that explicitly encode nonlinear mechanical behaviors into the microrobot architecture, alongside experimental validation of mechanical computation fidelity, noise tolerance, repeatability, and the robustness of decision-making under physiological fluctuations.

Finally, although the convergence of these directions could transform mechanically programmable structures into active biomedical platforms, clinical translation remains contingent on overcoming substantial barriers. These barriers include scalable manufacturing pipelines for heterogeneous microstructures, standardized protocols for biocompatibility and biodegradability assessment, real-time in vivo imaging and navigation systems with adequate spatial resolution, robust safety mechanisms that ensure retrieval or harmless clearance, and comprehensive regulatory frameworks governing autonomous operation within living organisms. Addressing these multifaceted requirements is essential before the structural advances surveyed in this review can achieve their full clinical potential.

## Figures and Tables

**Table 1:** Comparison of representative rigid, soft, and rigid–soft integrated microrobots (NR = not reported).

Category	Rigid Microrobots				Soft Microrobots				Rigid–Soft Integrated Microrobots			
**Key Structural Features**	Geometric Asymmetric Structures		Surface Asymmetric Structures		Predefined Deformation Structures		Dynamically Reconfigurable Structures		Spatially Heterogeneous Integration Structures		temporal stiffness modulation Structures	
**Representative microrobot**	ABF microrobot [54]	Tubular microrobot [94]	Janus microsphere [102]	Surface walker [109]	Bilayer microgripper [115]	Single-layer microrobot [125]	Kirigami microrobot [129]	Shape-morphing micromachine [138]	segmented shape-morphing micromachine [147]	Artificial musculoskeletal spider microrobot [157]	stiffness-tunable magnetorheological elastomers [162]	Stiffness- tunable Helixoft microrobot [173]
**Dimensions**	~47 μm	~50 μm	~5 μm	~2.8 μm	~100 μm	~250 μm	~1 mm wide	~10 μm per panel	~57.5 μm	~100–200 μm	~12.5 mm	diameters 300 μm^−1^ mm
**Material Composition**	InGaAs/GaAs/Cr/Ni/Au	Ti/Fe/Pt	Silica/Pt	Magnetic beads	PPF/pNIPPAm-AAc hydrogel	E-dent 400/MNPs	Light-responsiveLCNs	PMMA/Ti/Co/Al	IP-L photoresist/NIPAAm/AAm	SU-8 photoresist/BSA protein hydrogel	Magnetorheological fluid	Stainless-steel/silicone elastomer/NdFeB/PDMAA
**Effective Modulus/Stiffness**	NR	NR	NR	NR	kPa-MPa	NR	NR	NR	NR	SU-8 skeleton: 4.8 GPa	~10 kPa– 13 MPa	36 kPa–1500 kPa
**Actuation Mechanism**	Magnetic field	Chemical	Chemical	Magnetic field	Temperature + magnetic field	Magnetic field + NIR laser	Light	Magnetic field	ultrasound acoustic field	pH	Electromagnetic field	Magnetic field
**Locomotion Mode**	Corkscrew propulsion	Bubble-jet recoil propulsion	Translational	Walking/translation	Folding/gripping/releasing	Translational	Rolling	Flapping/hovering/turning/side-slipping	Folding/blooming/flapping	Bending/gripping	Grasping/releasing	Bending-steering/Axial motion
**Speed**	~1.8 μm/s	~275 μm/s	~9 μm/s	~6 μm/s	Deformation in ~seconds	~5 mm/s	1–5 mm/s	Deformation in ~seconds	Deformation in ~milliseconds	NR	NR	Axial advancement speed: 0–100 mm/s
**Force Output**	NR	NR	NR	NR	NR	NR	NR	6.54 nN−30.5 nN	NR	~34 μN	NR	NR
**Deformation/Stiffness-Switching Range**	No deformation	No deformation	No deformation	No deformation	2D-to-3D reversible folding	2D-to-tubular rolling/unrolling	Reversible petal bending-unbending	Reversible folding/bending/twisting	Deformation angle 0–75°	Reversible bending angle 0–23°	Maximum stiffness tuning ratio up to ~70-fold	>40-fold stiffness tuning/bending angle 118°
**Response Time**	NR	NR	NR	NR	Seconds to tens of seconds	NR	2 s	seconds	milliseconds	seconds	~20 ms	seconds
**Payload Capacity**	Polystyrene microspheres	Polystyrene microspheres	Superparamagnetic microbeads	NR	Micro-particles/cells	15.8 μg DOX per microrobot	NR	microbeads	NR	SU-8 microcube	Grasping macroscopic objects	25 μL doxorubicin anticancer drug solution
**Fabrication Method**	MBE thin-film growth	E-beam evaporation/magnetron sputtering	Self-assembly/magnetron sputtering	Self-assembly	Photolithography/micro-patterning	Focused UV light polymerization	Laser engraving	Electron-beam lithography/RIE	DLW	DLW	Component mixing/vacuum degassing/mold casting/thermal oven curing	Winding/assembly/
**Biological Model**	Deionized-water environment	H_2_O_2_ environment	H_2_O_2_ environment	Aqueous environment	In vitro cell culture (aqueous buffer)	In vitro cellular drug delivery	Human hand surface demonstration	NR	Deionized water/PBS buffer environment	Aqueous buffer environment	NR	Ex vivo porcine oviduct biopsy model/in vivo live Bama mini-pigs
**Biodegradability**	NR	NR	NR	NR	Partially degradable (hydrogel hydrolysis	NR	NR	NR	NR	NR	NR	NR
**Imaging/Tracking Method**	Optical	Optical	Optical	Optical	Optical/fluorescence microscopy	Real-time X-ray imaging	Optical	Optical	Optical	Optical	Optical	Optical/digital radiography imaging
**Level of Validation**	L1: locomotion & micro-object manipulation	L1: locomotion &micro-object manipulation	L1: locomotion & micro-object manipulation	L1: locomotion	L1: shape-morphing	L1: locomotion; L2: in vitro cell-level drug delivery	L1: locomotion	L1: locomotion &shape-morphing validation & inert microbead manipulation	L1: shape-morphing & inert-object manipulation	L1: shape-morphing & micro-object manipulation	L1: material-level characterization &centimeter-scale gripper proof-of-concept	L1: phantom lumen navigation; L3: ex vivo porcine oviduct biopsy; L4: in vivo live-pig bronchial drug-delivery intervention

**Table 2:** SWOT analysis of rigid, soft, and rigid–soft integrated microrobots for clinical translation.

	Rigid Microrobots	Soft Microrobots	Rigid–Soft Integrated Microrobots
**Strengths**	High propulsion efficiency and precise deterministic control; mature fabrication and modeling frameworks; strong force output for tissue penetration.	Excellent biomechanical compatibility; low tissue contact stress; ability to navigate tortuous and constricted anatomical geometries via deformation.	Synergistic unification of precision and compliance; task-phase-adaptive mechanics; potential for multifunctional compartmentalization.
**Weaknesses**	Severe mechanical mismatch with soft tissues; inability to deform through narrow lumens; limited biodegradability; risk of vascular occlusion and thrombosis.	Low force output limits tissue penetration; compromised motion precision and controllability; complex continuum mechanics modeling.	Unproven long-term interface reliability; fabrication complexity and low throughput; lack of mature theoretical frameworks for multi-physics coupled dynamics; potential multi-material toxicity.
**Opportunities**	Imaging-guided precision therapy; cell-level manipulation; telerobotic neurovascular interventions; thrombolytic therapy	Smart responsive drug release in GI and pulmonary tracts; minimally invasive navigation in narrow vessels; tissue engineering scaffolds with dynamic remodeling.	Personalized theranostic platforms; adaptive minimally invasive surgery; implantable devices requiring both structural support and tissue interfacing; embolization with shape adaptability.
**Threats**	Thrombosis/embolization; immune clearance; mechanical trauma; long-term retention of non-degradable components; field-induced heating.	Protein adsorption and biofouling; uncontrolled degradation or swelling; immune encapsulation; loss of structural integrity before mission completion; mucus entrapment.	Interface fatigue and delamination under cyclic physiological loading; multi-material toxicity from degradation products; complex regulatory pathways for heterogeneous devices; fragmentation-induced embolization.

## Data Availability

No new data were created in this review.

## Abbreviations

2D: two-dimensional; 3D: three-dimensional; ABF: artificial bacterial flagella; GLAD: glancing angle deposition; DLW: direct laser writing; MRI: magnetic resonance imaging; MOFs: metal–organic frameworks; PPF: polypropylene fumarate; pNIPAAm: poly (N-isopropylacrylamide); pNIPAAm-AAc: poly (N-isopropylacrylamide-co-acrylic acid); PPy: polypyrrole; PET: polyethylene glycol terephthalate; LMPAs: low-melting-point alloys; PDMS: poly (dimethylsiloxane); MREs: magnetorheological elastomers; PEG: polyethylene glycol; MNPs: magnetic nanoparticles; NIR: near-infrared; UV: ultraviolet; LCN: liquid crystal polymer network; PMMA: poly (methylmethacrylate); RIE: reactive-ion etching; AAm: acrylic ester; BSA: bovine serum albumin; PDMAA: polydimethylacrylamide; MBE: molecular-beam epitaxy.

## Appendix A

### Literature Search and Review Methodology

The literature corpus for this review comprises peer-reviewed microrobots studies retrieved from Web of Science, PubMed, Scopus, and IEEE Xplore, published primarily between 2000 and 2026. Inclusion was limited to works that explicitly advance one of the three structural paradigms systematically examined in this review, namely rigid architectures, soft architectures, and rigid–soft integrated systems, within a biomedical context. Representative studies were prioritized based on their pioneering significance in establishing or transitioning structural paradigms, the depth of their experimental validation, and their contribution to balanced structural diversity across categories. Works targeting non-biomedical applications, actuation mechanisms without structural design implications, or material syntheses lacking architectural innovation were excluded from the central narrative.
